# Global report on preterm birth and stillbirth (7 of 7): mobilizing resources to accelerate innovative solutions (Global Action Agenda)

**DOI:** 10.1186/1471-2393-10-S1-S7

**Published:** 2010-02-23

**Authors:** Craig E Rubens, Michael G Gravett, Cesar G Victora, Toni M Nunes, the GAPPS Review Group

**Affiliations:** 1 Global Alliance to Prevent Prematurity and Stillbirth, an initiative of Seattle Children's, Seattle, Washington, USA; 2 Department of Pediatrics at University of Washington School of Medicine, Seattle, Washington, USA; 3 Department of Obstetrics and Gynecology, University of Washington, Seattle, WA USA; 4 Universidade Federal de Pelotas, Pelotas 96001-970, Brazil

## Abstract

**Background:**

Preterm birth and stillbirth are complex local and global health problems requiring an interdisciplinary approach and an international commitment. Stakeholders developed recommendations for a Global Action Agenda (GAA) at the 2009 International Conference on Prematurity and Stillbirth. The primary goal of this GAA is to forge a collaborative effort toward achieving common goals to prevent preterm birth and stillbirth, and to improve related maternal, newborn, and child health outcomes.

**Conference participants:**

GAPPS co-convened this four-day conference with the Bill & Melinda Gates Foundation, March of Dimes, PATH, Save the Children, UNICEF and the World Health Organization. Participants included about 200 leading international researchers, policymakers, health care practitioners and philanthropists. A near-final draft of this report was sent three weeks in advance to help co-chairs and participants prepare for workgroup discussions.

**Global Action Agenda:**

Twelve thematic workgroups, composed of interdisciplinary experts, made recommendations on short-, intermediate-, and long-term milestones, and success metrics. Recommendations are based on the following themes: (1) advance discovery of the magnitude, causes and innovative solutions; (2) promote development and delivery of low-cost, proven interventions; (3) improve advocacy efforts to increase awareness that preterm birth and stillbirth are leading contributors to the global health burden; (4) increase resources for research and implementation; and (5) consider ethical and social justice implications throughout all efforts.

**Summary:**

The conference provided an unprecedented opportunity for maternal, newborn and child health stakeholders to create a collaborative strategy for addressing preterm birth and stillbirth globally. Participants and others have already completed or launched work on key milestones identified in the GAA. Updates will be provided at www.gapps.org.

## Background

Despite the significant global burden of preterm birth and stillbirth, these issues have attracted remarkably little attention and investment. Some of this void can be attributed to a shortage of adequate data. As multiple causes and pathways contribute to preterm birth and stillbirth, a comprehensive, interdisciplinary approach is needed to prevent these outcomes.

The Global Action Agenda (GAA) highlights the need for a collaborative, international commitment for the discovery, development, and delivery of cost-effective interventions. Global advocacy efforts are critical to increase visibility and resources for these issues. All efforts must be guided by ethical and social justice principles. These issues are discussed in the first six articles of this global report [1-6].

In May 2009, the Global Alliance to Prevent Prematurity and Stillbirth (GAPPS), an initiative of Seattle Children's, co-convened the 2009 International Conference on Prematurity and Stillbirth with the Bill & Melinda Gates Foundation, March of Dimes, PATH, Save the Children, UNICEF and the World Health Organization. All participants shared a common goal: to improve maternal, newborn and child health globally.

### Goals of the International Conference in Seattle, WA, USA

The three primary goals set for workgroups during the conference are outlined below:

• Develop a roadmap of short-term, intermediate, and long-term milestones, including an international research agenda that would lead to new interventions

• Identify the most successful current interventions to improve maternal, fetal, newborn and child health outcomes

• Set the stage for policy action among global stakeholders 

### Overview of participants

An interdisciplinary group of 185 experts participated in this invitation-only meeting. Participants represented 35 countries, with diverse participation from low-, middle-, and high-income countries. They included researchers, healthcare practitioners, UN and government agencies, nonprofits, policymakers, and funders. Two dozen co-chairs led an intensive four-day effort to develop a comprehensive and coordinated action strategy to improve pregnancy outcomes.

### Format of working conference

All participants received a nearly completed draft of articles 1 through 6 of this report three weeks prior to the conference, as well as summary presentations at the beginning of the meeting by the GAPPS team of investigators. This information provided a solid foundation that helped make workgroup discussions highly productive and accelerated the creation of these recommendations for a Global Action Agenda (GAA). Many participants also provided invaluable feedback on the report.

The conference program was composed of an opening session, plenary sessions, and workgroup meetings and presentations. A dozen thematic workgroups included an average of 15 interdisciplinary stakeholders to ensure vigorous discussion and foster continued collaboration beyond the conference. An archived Webcast of the conference is available at www.gapps.org.

Each workgroup was led by two co-chairs with specific expertise in the given topic.

## Summary of workgroup recommendations for the Global Action Agenda

Workgroups identified overarching goals and several key outputs for the GAA. In addition, they identified specific milestones, set to a uniform timeline, and metrics of evaluation that corresponded with each output. (See Tables 1, 2, 3, 4, 5, 6, 7, 8, 9, 10, 11, 12.)

**Table 1:** Normal Gestational Biology A GLOBAL ACTION AGENDA ON PRETERM BIRTH AND STILLBIRTH

**Overarching Goal:** To gain comprehensive knowledge of the biology and regulation of human gestational biology in order to identify pathways and critical junctures to facilitate prediction and prevention of preterm birth and stillbirth					
	Milestones*				
		
**Output**	**Post-Conference (by 2010)**	**Short-Term (by 2012)**	**Intermediate (by 2015)**	**Long-Term (beyond 2015)**	**Success Metrics**

**A. Define phenotype of normal pregnancy**	1. Systematic review of existing knowledge 2. Develop "best guess" phenotype of normal gestation	3. Prospective tissue repository of normal gestation 4. Utilize "systems biology" technology 5. Identify critical biomarkers, including those governing parturitional stages and biomarkers of placentation, fetal growth and development	6. Develop "lateral flow" multi-analyte diagnostic platforms 7. Begin application of diagnostic tools in LMICs	8. Validate multi-analyte platforms for both HICs and LMICs	• Comprehensive atlas of normal human gestation • Web-based tool for providers and patients
**B. Develop animal and in-silico models of normal parturition**	1. Systematic review of existing knowledge	2. Explore and develop animal models including genetically altered mice, non-human primates, others 3. Computer modeling 4. Integrate modeling with animal models	5. Validate models 6. Predict outcome based on modeling	7. Use models to predict therapeutic targets and treatments	• Improved animal models to identify key regulatory steps • Enhanced use of in-silico models
**C. Define regulators and mechanisms governing stages of parturition**	1. Systematic review of existing knowledge	2. Identify critical biomarkers governing parturitional stages and transitions	3. Identify potential therapeutic targets for treatment of PTL 4. Develop therapeutic interventions based upon selected targets 5. Collaborate with Intervention Development to prioritize potential therapeutic interventions	6. Develop therapeutic interventions based upon selected targets and scaling feasibility	• Identify novel therapeutic and diagnostic targets • Web-based tool for providers and patients

*Milestones are to be reached by no later than December of the year indicated.

**Table 2:** Abnormal Gestational Biology A GLOBAL ACTION AGENDA ON PRETERM BIRTH AND STILLBIRTH

**Overarching Goal:** To understand the mechanisms contributing to preterm birth and stillbirth, with emphasis upon infectious, genetic, and environmental factors, abnormal placental vascular development, and early gestational events					
	Milestones*				
		
**Output**	**Post-Conference (by 2010)**	**Short-Term (by 2012)**	**Intermediate (by 2015)**	**Long-Term (beyond 2015)**	**Success Metrics**

**A. Determine causes and differential susceptibility to infection, and maternalrfetal immune response associated with PTB and SB utilizing high-dimensional systems biology approaches**	1. Identify existing cohorts to characterize the pregnancy "biome"	2. Treat existing known infectious causes of PTB/SB 2. Establish cohort to characterize pregnancy "biome" 3. Characterize human vaginal microbiome	5. Identify polymorphisms and immunoregulatory genes associated with PTB/SB 6. Utilize systems biology to identify non-invasive biomarkers for PTB/SB 7. Study vaginal and cervical mucosal immunity and the biology of the microbial flora	8. Validate models to assess intervention strategies 9. Study short-and long-term conseguences of inflammation on fetal origin of adult disease and neurodevelopmental outcome	• Cost-effective interventions to reduce morbidity/mortality associated with inflammation- induced prematurity
**B. Determine causes of vascular mal-adaptation resulting in abnormal uteroplacental perfusion, fetal growth restriction and abruption associated PTB and SB utilizing high-dimensional systems biology approaches**	1. Increase grant RFAs by national and international research funding agencies	2. Study origins of spiral artery adaptation 3. Study genetic and environmental influences on vasculopathy 4. Develop models for vascular pathology, including endometrial modifications	5. Develop cost-effective interventions to promote normal placentation	6. Study short-and long-term conseguences on fetal origin of adult disease and neurodevelopmental outcomes	• Cost-effective interventions to reduce morbidity/mortality associated with vascular associated prematurity linked to abnormal uteroplacental vasculature
**C. Determine if preconceptual and/or antenatal micronutrient exposure contributes to PTB/SB**	1. Systematic review of available evidence	2. Cohort studies to confirm associations	3. RCTs of micronutrient support or environmental modification 4. Assess translational feasibility	5. Specific trials for LMICs	• Identification of cost-effective micronutrient interventions to reduce PTB/SB

*Milestones are to be reached by no later than December of the year indicated.

**Table 3:** Genetic and Environmental Factors A GLOBAL ACTION AGENDA ON PRETERM BIRTH AND STILLBIRTH

**Overarching Goal:** To determine and reduce the role of genetics, the environment, and their interactions on the burden of preterm birth and stillbirth					
	Milestones*				
		
**Output**	**Post-Conference (by 2010)**	**Short-Term (by 2012)**	**Intermediate (by 2015)**	**Long-Term (beyond 2015)**	**Success Metrics**

**A. Characterize genetic risks for PTB/SB and identify potentially modifiable environmental influences, especially for LMICs**	1. Systematic review of existing knowledge 2. Identify and engage potential funding agencies 3. Assess existing cohorts	4. Develop cohorts in LMICs 5. Develop standardized phenotype definitions and collection protocols 6. Generate RFAs 7. Initiate large scale GWAS 8. Initiate microbiome studies 9. Initiate epigenetic studies and gene:environment studies	10. Develop geographic and culturally valid measures of environmental, genetic, and nutrient risks	11. Population-appropriate intervention trials of modifiable genetic influences	• Contribute to MDG 4 • Standardized protocols and phenotype definitions
**B. Intensively characterize the "envirome" (xenobiotics, microbiomes, environmental influences) relative to the global risks for PTB/SB**	1. Systematic review of existing knowledge 2. Identify and engage potential funding agencies	3. Utilize in-vitro models for high-throughput screening of xenobiotics and PTB/SB 4. Develop appropriate bio-informatics infrastructure 5. Generate RFAs	6. Epidemiologic studies to assess associations of environmental exposure and PTB/SB 7. Initiate clinical trials of modifiable environmental risk factors	8. Develop exportable screening tools for environmental risks 9. Clinical trials of modifiable environmental risk factors	• Ethnically and geographically valid measures for nutritional stress • Established population-attributable risks to exposures

*Milestones are to be reached by no later than December of the year indicated.

**Table 4:** Epidemiology of Preterm Delivery A GLOBAL ACTION AGENDA ON PRETERM BIRTH AND STILLBIRTH

**Overarching Goal:** To improve collection, analysis, interpretation, and application of epidemiological data as a basis for interventions to reduce preterm birth					
	Milestones*				
		
**Output**	**Post-Conference (by 2010)**	**Short-Term (by 2012)**	**Intermediate (by 2015)**	**Long-Term (beyond 2015)**	**Success Metrics**

**A. Improve descriptive epidemiology of PTB**	1. Create expert group to define phenotypes of PTB 2. Develop tools/algorithm for gestational age assessment relevant to community-based resources 3. Develop standard definitions for impairment outcomes	4. Selective testing of gestational age assessment tools 5. Establish surveillance sites in LMICs 6. Promote incorporation of phenotype definitions into existing perinatal databases in HICs	7. Prepare global, country-specific report of PTB and trends		• Peer review publications • Improved understanding of country specific etiologies of PTB • Accurate global charts of PTB rates
**B. Improve analytical (riskfactor identification) epidemiology**	1. Systematic review of risk factors for PTB in different settings (Global report on preterm birth and stillbirth)	2. Assess verbal autopsy to measure risk factors 3. Promote phenotype definition 4.Incorporate phenotype definitions in global databases	5. Prepare global, country-specific report of PTB and trends		• Peer review publications • Improved understanding of country-specific etiologies of PTB • Accurate global charts of PTB rates
**C. Strengthen data collection and analysis capacity to inform health policy**	1. Identify existing networks, stakeholders in PTB research 2. Develop structure for collaborating and disseminating resources among networks and stakeholders 3.Raise political awareness	4. Investigate low-tech data entry resources 5. Train personnel in data collection at local and regional levels	6. Cost-effective surveillance	7. Cost-effective surveillance	• Adoption of standardized collection and analysis tools • Resource infrastructure that can be shared between networks and stakeholders

*Milestones are to be reached by no later than December of the year indicated.

**Table 5:** Stillbirth Epidemiology A GLOBAL ACTION AGENDA ON PRETERM BIRTH AND STILLBIRTH

**Overarching Goal:** Stillbirths are an important indicator of women's health, and accurate collection of data will help influence health care providers and policy makers to improve maternal and child health					
	Milestones*				
		
**Output**	**Post-Conference (by 2010)**	**Short-Term (by 2012)**	**Intermediate (by 2015)**	**Long-Term (beyond 2015)**	**Success Metrics**

**A. Ensure the collection of comparable data of high quality, and build capacity**	1. Catalogue current efforts of facility-and population-based data collection 2. Coordinate efforts to improve measurement in large-scale population-based surveys	3. Consensus meeting to identify uniform minimal data collection on all pregnancies (refine in parallel with classification system) 4. Create internet-based resources and tools for accurate measurement	5. 100% of countries have a national empirical estimate of SB rate 6. Establish 100% country compliance with at least two metrics of SB		• Defined distribution of risk factors of SB from these minimal datasets, especially in high SB mortality countries • International comparisons
**B. Develop uniform classification for stillbirths**	1. Create network for classification of SB	2. Develop uniform classification system (refine in parallel with uniform minimal data set) 3. Test validity against existing standards in HICs and in LMICs			• Adoption of classification system to allow international comparisons
**C. Develop targeted and in-depth population based studies**		1. Initiate population-based studies, with control groups, in regions with high SB mortality 2. Identify specific etiologies for SB	3. Institute regional, population-specific intervention trials		• Defined distribution of risk factors of SB, especially in high SB mortality countries
**D. Inform evidence-based policies and interventions**	1. Identify potential funding sources	2. Establish infrastructure for data collection and analysis 3. Train personnel in data collection at local and regional levels	4. Define and evaluate data quality indicators		• Implementation of evidence-based policies/interventions

*Milestones are to be reached by no later than December of the year indicated.

**Table 6:** Intervention Development A GLOBAL ACTION AGENDA ON PRETERM BIRTH AND STILLBIRTH

**Overarching Goal:** Generate knowledge to develop new capacities and strengthen existing capacities to improve birth outcomes					
	Milestones*				
		
**Output**	**Post-Conference (by 2010)**	**Short-Term (by 2012)**	**Intermediate (by 2015)**	**Long-Term (beyond 2015)**	**Success Metrics**

**A. Set and disseminate research priorities**	1. Complete formal CHNRI process and manuscript	2. Publish manuscript of CHNRI analysis 3. Issue RFA based on research priorities	4. Update research priority exercise 5. Update Global report on preterm birth and stillbirth, and research priorities 6. Issue second RFA based on revised research priorities	7. Promote continuing feedback between research priority exercise and results from new research, with the involvement of funders and policymakers	• Manuscript developed for publication • Global report on preterm birth and stillbirth, and research priorities updated (5 years)
**B. Complete prioritized research and share results**	1. Obtain commitment from funders and buy-in from stakeholders on finalized research agenda	2. Allocate funding on the basis of the RFA 3. Complete prioritized research 4. Results inform advocacy stakeholders to build political will for interventions	5. Research leads to new interventions that are implemented at large scale in a few countries 6. Research informs each stage along the continuum: Discovery, Development, Delivery and Advocacy 7. New round of funding allocation based on revised research agenda 8. Complete revised prioritized research	9. Interventions resulting from the research initiative are implemented at scale in large numbers of countries 10. Second round of research leads to new interventions that are implemented at large scale in a few countries	• RFAs issued to address priority research interventions • Priority research completed • Successful research implemented at scale
**C. Strengthen research capacity**	1. Obtain commitment from funders for an initiative to build on-site capacity for intervention development and clinical trials 2. Develop database of active research projects on preterm birth and stillbirth	3. Establish research network (2010) 4. Establish and link regional Centers for Excellence in LMICs (2011)	5. Strengthened research capacity contributes to improvements in research and to the development of new, locally-relevant interventions	6. Donor investments in research in LMICs and HICs.and research capacity building in LMICS, are significantly expanded, resulting in a shift of the global research divide	• Network of SB/PTB researchers established • Systems of regional centers of excellence established in LMICs • Donor investments to research institutions 50:50 LMICs/HICs

*Milestones are to be reached by no later than December of the year indicated.

**Table 7:** Prioritization of Interventions for Scaling Up A GLOBAL ACTION AGENDA ON PRETERM BIRTH AND STILLBIRTH

**Overarching Goal:** Reduce stillbirths and mortality due to preterm birth through development and application of dynamic processes that engages stakeholders for prioritization of evidence-based and context-specific interventions, delivered with high coverage and equity					
	Milestones*				
		
**Output**	**Post-Conference (by 2010)**	**Short-Term (by 2012)**	**Intermediate (by 2015)**	**Long-Term (beyond 2015)**	**Success Metrics**

**A. Advocate use of evidence in prioritization among stakeholders**	1. Among stakeholders, advocate the use of evidence for developing context-specific intervention priorities 2. Involve local stakeholders responsible for implementation in prioritization process 3. Inform donors	4. Contribute to the development of a generalized process for intervention priority-setting 5. Identify opportunities for using priority-setting tools in LMICs and support their implementation	6. Contribute to continuous refinement of prioritization processes and intervention tools	7. Intervention prioritization processes are mainstreamed in policy decisions throughout the world	• Increase in stakeholders utilizing evidence-based processes of prioritizing interventions to reduce preterm births and stillbirths
**B. Ensure inclusion of preterm birth and stillbirth interventions into existing prioritization processes**	1. Utilize opportunity to raise profile of these two issues in relation to MDGs4and5 2. GAPPS to support efforts led by JHSPH to develop LiST tool, as this may be adapted to PTB and SB	3. Enhance decision support tools (e.g. LiST, CHOICE, MBB) to address potential effects on mortality and cost implications for scaling up interventions directed at stillbirths and preterm births 4. Develop process for identifying factors outside scope of existing decision support tools and incorporating these factors in estimates	5. Decision support tools that incorporate stillbirths and preterm deliveries are disseminated in LMICs	6. All countries use decision making tools	• Increased, equitable coverage of selected interventions that are appropriate to the context that they are applied
**C. Select best intervention candidates for scale-up in health facilities**	1. Prioritization for scale-up in areas with moderate to high utilization/access to health care facilities for antenatal care and delivery	2. Scale up evidence-based intervention use within facilities providing maternal and neonatal care 3. Implement and scale up interventions that are appropriate to context and resources 4. Increase equity of access to facilities and their interventions	5. Facility-based interventions are scaled up in all appropriate areas in LMICs	6. Newly developed and existing facility-based interventions are regularly subjected to prioritization exercises to take into account changes in technology, demographics, burden of disease and costs	• Successful development and utilization of advanced processes, methods and tools used to prioritize facility interventions that leads to high coverage and contributes to improving population health
**D. Select best intervention candidates for scale-up in communities and homes**	1. Prioritization for scaling up interventions in areas with low access to health care facilities	2. Scale up home-based care that is context- and resource-appropriate 3. Strengthen capacity for community case management of pregnancy and neonatal health to expand list of interventions that can optimally be scaled up	4. Community-basedinterventions are scaled up in all appropriate areas in LMICs	5. Newly developed and existing community based interventions are regularly subjected to prioritization exercises to take into account changes in technology, demographics, burden of disease and costs	• Successful development and utilization of advanced processes, methods and tools used to prioritize community interventions that leads to high coverage and contributes to improving population health

*Milestones are to be reached by no later than December of the year indicated.

**Table 8:** Community-Based Strategies and Constraints A GLOBAL ACTION AGENDA ON PRETERM BIRTH AND STILLBIRTH

**Overarching Goal:** To achieve the maximum reduction in stillbirths and neonatal deaths due to preterm births by implementing effective community-based approaches at high coverage within the continuum of maternal and newborn care. We aim to achieve by 2020, a one-third reduction in stillbirths and two-thirds reduction in neonatal mortality due to preterm birth in 68 high mortality countries					
	Milestones*				
		
**Output**	**Post-Conference (by 2010)**	**Short-Term (by 2012)**	**Intermediate (by 2015)**	**Long-Term (beyond 2015)**	**Success Metrics**

**A. Deploy, strengthen and sustain community health workers (CHWs) at scale**	1. Endorse community-based intervention packages as essential to addressing these problems at scale 2. Strengthen the Global report on preterm birth and stillbirth with evidence-based community mechanisms (done) 3. Map current packages delivered by community-based MNH workers in different countries	4. Create global consensus on community-based intervention packages for scale-up 5. Study process of current community-based interventions to expand evidence base for delivery and scale-up 6. Introduce community-based packages in at least 10 countries	7. Introduce community-based packages in remaining Countdown countries	8. All LMICs have sufficient numbers of well-trained, eguipped and supervised CHWs to deliver community interventions at high and eguitable coverage	• Marked increase in coverage of community-based interventions • Reduced neonatal mortality and stillbirth rates
**B. Build capacity of community to identify, promote and monitor actions**	1. Start review of successful experiences with community based problem-identification and monitoring	2. Support community-based health information systems in a few countries	3. Scale up community-based information systems in a large number of countries	4. All LMICs have high coverage of community-based information systems	• Marked increase in the availability of health data at community level in all countries
**C. Promote and enhance support structures for CHWs and communities**	1. Identify key elements of support system for CHWs and community-based interventions (e.g., training, supervision, drug supplies, and educational materials) 2. Strengthen the Global report on preterm birth and stillbirth with evidence-based community delivery mechanisms	3. Obtain consensus on intervention packages that can be delivered at the community level and scaled up, initially in a few countries 4. Advocate for strengthening the support systems for CHWs and other community-based MNCH interventions	5. Scale up community-based interventions to remaining countries 6. Analyze evidence and experiences of interventions delivered in short-term	7. Ensure the sustainability of CHWs and community-based systems	• Reduction in the stillbirth rate by one-third and newborn mortality due to preterm birth by two-thirds

*Milestones are to be reached by no later than December of the year indicated.

**Table 9:** Facility-Based Strategies and Constraints A GLOBAL ACTION AGENDA ON PRETERM BIRTH AND STILLBIRTH

**Overarching Goal:** By 2015 all community members will have timely access to effective, affordable and high-quality facility-based MNCH care provided by informed and responsive HWs as part of an integrated and equitable system to reduce perinatal mortality and morbidity					
	Milestones*				
		
**Output**	**Post-Conference (by 2010)**	**Short-Term (by 2012)**	**Intermediate (by 2015)**	**Long-Term (beyond 2015)**	**Success Metrics**

**A. Obtain funding dedicated to a prioritized research agenda**	1. Develop a comprehensive conceptual framework: accountability, referral, organization, regulatory interventions 2. Develop a complete set of research questions and submit these to the CHNRI process, producing a manuscript with priorities for research	3. Develop protocols answering top questions 4. Obtain funding for priority research studies 5. Build in-country research capacity 6. Conduct research and disseminate results	7. Research findings aredisseminated and effectively incorporated into health policy	8. Research agenda is regularly updated to incorporate changes in technology, demographics, burden of disease and costs	• Priority research items receive funding • Manuscript developed for publication
**B. Provide quality care for all community members attending health facilities**	1. Define packages and integrate with existing ones 2. Set standards 3. Increase the availability of existing packages 4. Get stakeholder buy-in 5. Develop capacity to implement in-country	6. Conduct situation analysis at country level 7. Create district map of availability of interventions (2010) 8. Choose and estimate the cost of solutions 9. Identify constraints (e.g., capacity, political will) 10. Create intervention plan (2011)	11. Implementation and scale up of facility interventions in a large number of countries	12. Ongoing: Evaluate, monitor indicators	• Defined country-level packages • Mapped gaps in facility- based care • Strategy implementation • Monitoring and evaluation of quality indicators
**C. All facilities provide core packages of MNCH services at first and referral levels; Quality facility-based care is accessible to all mothers, newborns and children in a timely manner**	1. Complete GAPPS review of prioritized facility-based interventions	2. Define package for each level 3. Conduct situation analysis 4. Identify measurable targets 5. Establish clear policy for no point-of-care payment 6. Use public-private partnership to make private MNC services available to all at no cost to families (e.g., vouchers) 7. Provide community support in using services and community ownership of facilities 8. Secure funding 9. Engage civil society to ensure accountability	10. Existing facilities provide the appropriate package of care in all countries: 1) policy on the importance of facilities for achieving MDGs 4 and 5; 2) human resources - task shifting, capacity building, deployment motivation; equipment, supplies, maintenance, infrastructure; managerial - 24/7 services; funding 11. Government covers family costs in accessing care (e.g., transport)	12. Establish new facilities to ensure adherence to benchmarks (population/ facility ratio) Note: All 5 components from intermediate-term milestones apply here as well 13. Improve transportation where needed 14. Facilities establish close communities in excess of population-facility ratio benchmarks	• Proportion of facilities meeting defined standards • Facility to population ratio (overall, by geographic regions and disaggregated by underserved populations) • Proportion of facilities where there is no point of care fee • Proportion of facilities where the governing body has adequate community leadership

*Milestones are to be reached by no later than December of the year indicated.

**Table 10:** Advocacy and Policy A GLOBAL ACTION AGENDA ON PRETERM BIRTH AND STILLBIRTH

**Overarching Goal:** Key actors allocate sufficient resources and support policies, programs, and actions at the global, regional, national, and community levels to ensure safe full-term pregnancies and healthy newborns					
	Milestones*				
		
**Output**	**Post-Conference (by 2010)**	**Short-Term (by 2012)**	**Intermediate (by 2015)**	**Long-Term (beyond 2015)**	**Success Metrics**

**A. Increase funding for research on the scope, causes, consequences, interventions, and scaling up of interventions for preterm birth and stillbirth**	1. Identify champions (recruit and mobilize) 2. Develop fact sheet that presents need, gap and benefits of research on PTB/ SB and link to MDGs4&5 (RMNCH)	3. Mobilizing professional organizations 4. Educating decision makers 5. Identify and prioritize funding targets among national governments, public-private partnerships, international nonprofit agencies and large research funding donors			• Number of research projects on PTB and SB • Funds being expended on PTB and SB • Completed research projects • Statements from donor entitites (from H8, for example) that mention PTB/SB as an issue that needs attention • Number of professional associations at a global and national level that call for increased research funding
**B. Increase awareness of the magnitude, impact, and opportunities to reduce and prevent preterm birth and stillbirth, as they relate to the accomplishments of the MDGs**	1. Develop key messages, fact sheets and success stories 2. Educate MNCH community internally 3. Create an advocacy/ communications network	4. Develop in-country community outreach, including men and women, and community influences 5. Outreach to policymakers through regional and global forums and partnerships	6. Create a global awareness campaign		• Number of people who publicly speak about personal experiences with PTB and SB • Media coverage (media as a proxy) • Surveys, polls • DHS incorporates guestions on PTB/ SB • Incorporation of PTB/SB messages into materials of MNCH agencies, initiatives, partnerships • Number of policymaker statements that include PTB and SB messages
**C. Build financial and political support for scaling up a core set of evidence-based, effective interventions for preventing/ managing PTB and SB, and integrate into national policies and guidelines**	1. Identify set of universal priorities based on existing information	2. Recruit and mobilize champions 3. Present and disseminate global reports and country studies on PTB and SB 4. Define and promote policy proposals 5. Focus event			• Number of regional/national plans that incorporate PT and SB with funds allocated • Number of partnerships that incorporate PTB and SB • Dissemination of credible, authorized studies to decisionmakers • Number of champions and supporters mobilized • Funding allocations for MNCH by donor countries

*Milestones are to be reached by no later than December of the year indicated.

**Table 11:** Ethics and Social Justice A GLOBAL ACTION AGENDA ON PRETERM BIRTH AND STILLBIRTH

**Overarching Goal:** To help inform an ethically responsible and culturally appropriate response to the global burden of preterm and stillbirth					
	Milestones*				
		
**Output**	**Post-Conference (by 2010)**	**Short-Term (by 2012)**	**Intermediate (by 2015)**	**Long-Term (beyond 2015)**	**Success Metrics**

**A. Identify the range of ethical or social justice considerations that arise along the research pathway, from definitions to discovery, development and delivery**	1. Establish standing working group on ESJ in preterm birth and stillbirth 2. Publish "Points to Consider," reflecting the most pressing ethical considerations identified by the work group, across the research pathway	3. Publish special issue of Indian Journal of Medical Ethics devoted to ethical issues in prematurity and stillbirth, from Indian perspectives	4. Identify an appropriate journal to publish African perspectives on ethical issues in prematurity and stillbirth (possibly Developing World Bioethics)		• Increased awareness and discussion of the ethics and social justice issues as evidenced by inclusion in peer-reviewed journals
**B. Engage scientists and key stakeholders regarding the ethical and social justice considerations identified above**	1. Invite scientists and other stakeholders involved in the conference to submit ethical issues encountered in the field	2. Identify scientific and professional conferences to present papers and panels on ethical issues surrounding preterm and stillbirth (e.g., PAS and Global Forum)			• Increased dialogue among scientists and bioethics of the ethics and social justice issues as evidenced by inclusion in scientific conferences
**C. Set a research agenda to address gaps in ethical guidance, policy, and cross- cultural understanding of the ethical issues surrounding the global burden of prematurity and stillbirth**	1. ESJ work group to identify a list of priority normative and empirical research questions surrounding the ethical and cross-cultural issues in preterm and stillbirth 2. Submit panel proposal to the 2010 World Congress in Bioethics, devoted to papers on "ethical considerations in the global burden of prematurity and stillbirth"	3. Treuman Katz Center for Pediatric Bioethics July 2010 to devote annual conference to'Ethical Issues at the Beginning of Life: Prematurity and Neonatology" 4. Seek funding for satellite meeting to reconvene the ESJ workgroup in July 2010 5. Seek funding to support empirical studies in India and Zambia on cross-cultural experiences of women surrounding preterm and stillbirth 6. Commission law review article to conduct a health and human rights analysis of reproductive decision-making surrounding preterm birth and stillbirth measurement 7. Facilitate the development of better measurements for stillbirth within the global burden of disease that address ethical concerns regarding stillbirth measurements	8. Identify additional country sites to conduct empirical studies on cross-cultural experiences of preterm and stillbirth, as well as additional factors identified in previous studies		• Improved understanding of the ethical, social, political, and cross- cultural ethical issues surrounding preterm birth and stillbirth, demonstrated in both normative and empirical research findings

*Milestones are to be reached by no later than December of the year indicated.

**Table 12:** Resource and Development Funding A GLOBAL ACTION AGENDA ON PRETERM BIRTH AND STILLBIRTH

**Overarching Goal:** Improve coordination and increase global and national funding for PTB and SB within the Reproductive Maternal Newborn Child Health (RMNCH) context					
	Milestones*				
		
**Output**	**Post-Conference (by 2010)**	**Short-Term (by 2012)**	**Intermediate (by 2015)**	**Long-Term (beyond 2015)**	**Success Metrics**

**A. Effective international RMNCH leadership to influence global health initiatives**	1. Develop strategies to elevate PTB and SB on the global RMNCH agenda Engage UN agencies and donors 2. H8 and other representatives 3. H8 meeting to include additional agenda item: consider most effective leadership and funding strategies to advance RMNCH agenda Engage governments - G8 meeting in July in Italy: support strong leadership and funding to advance the RMNCH agenda - Help promote issue with Obama administration in preparation for G8 and G20 - Engage Obama administration regarding their evolving Global Health Initiative - G20 - support strong leadership and funding to advance the RMNCH agenda	4. High-level agreement that RMNCH agenda needs to be strengthened (by 2010) 5. RMNCH global task force established that includes PTB and SB (by 2010) 6. Successfully building toward $ 10B annual fund for RMNCH interventions – ties to MDG #4-5 (by 2010) 7. Development of national plans and allocation of domestic and non- domestic resources to execute against plans			• RMNCH included in approval proposals to the Global Fund and other funding mechanisms
**B. Catalyze, facilitate, leverage and provide targeted support to engage national governments to test and/ or scale up effective interventions**	1. Assure that funding is available to assess current situation in 10 countries 2. Foster harmonization and alignment among development partners	3. Align national strategies with evidence-based approaches (by 2011) 4. Identify appropriate and effective intervention packages that can be scaled up in selected high-mortality countries (by 2011) 5. Engage with 10 high-mortality countries to implement MNCH strategies (by 2011) 6. Increase national capacity for spending domestic resources as well as accessing international resources through mechanisms such as debt relief (by 2011) 7. Influence country by providing funding to national civil society to hold countries accountable (by 2011)	8. Accelerate and improve implementation of interventions throughout health system 9. Harness the in-country private sector to contribute to increase coverage of key interventions 10. Support capacity building for measurement and evaluation 11. Increased integration and eguitable coverage of key, effective interventions into country within 5 years		• Increased integration and eguitable coverage of key, effective interventions into country within 5 years • Increased funding and resources from national governments • Assessment and evaluation data documenting reduced mortality
**C. Accelerate progress in the Discovery, Development, Delivery (3D) cycle**	1. Encourage funders to establish integrated 3D teams 2. Publish a model for integrated 3D process 3. Support a milestone related process for funding to bring promising discoveries to scale	4. Strengthen funding mechanisms to support 3D research in LMICs (by 2010)			• Survey funding organizations and determine which have established integrated 3D teams Publish a model for integrated 3D process Increased funding for 3D research in LMICs that are developing capacity

*Milestones are to be reached by no later than December of the year indicated.

Post-conference, co-chairs and other participants were encouraged to review a draft of the GAA to ensure all salient points were included and provide additional suggestions, including lead agencies and core team members. Recommendations included in the GAA reflect the consensus reached at the meeting. Additional recommendations received from individuals post-conference have not been included unless vetted by the workgroup.

Most milestones are set to be achieved by 2012, and the latest by 2015 to correspond with the United Nations Millennium Development Goals (MDGs). This is a living document that will be updated at least once annually. It will also be available at www.gapps.org. Below is a summary of the overarching goals and outputs identified by the twelve thematic workgroups.

### 1. Normal gestational biology 

#### Overarching goal

To gain comprehensive knowledge of the biology and regulation of human gestational biology in order to identify pathways and critical junctures to facilitate prediction and prevention of preterm birth and stillbirth

#### Key outputs identified by this workgroup

• Define phenotype of normal pregnancy

• Develop animal and in-silico models of normal parturition

• Define regulators and mechanisms governing stages of parturition

### 2. Abnormal gestational biology

#### Overarching goal

To understand the mechanisms contributing to preterm birth and stillbirth, with emphasis upon infectious, genetic, and environmental factors, abnormal placental vascular development, and early gestational events

#### Key outputs identified by this workgroup

• Determine causes and differential susceptibility to infection, and maternal:fetal immune response associated with preterm birth and stillbirth utilizing high-dimensional systems biology approaches

• Determine causes of vascular mal-adaptation resulting in abnormal uteroplacental perfusion, fetal growth restriction and abruption associated with preterm birth and stillbirth utilizing high-dimensional systems biology approaches

• Determine if preconceptual and/or antenatal micro-nutrient exposure contributes to preterm birth and stillbirth

### 3. Genetic and environmental factors

#### Overarching goal

To determine and reduce the role of genetics, the environment, and their interactions on the burden of preterm birth and stillbirth

#### Key outputs identified by this workgroup

• Characterize genetic risks for preterm birth and stillbirth, and identify potentially modifiable environmental influences, especially for LMICs

• Intensively characterize the "envirome" (xenobiotics, microbiomes, environmental influences) relative to the global risks for preterm birth and stillbirth

### 4. Epidemiology of preterm delivery

#### Overarching goal

To improve collection, analysis, interpretation, and application of epidemiological data as a basis for interventions to reduce preterm birth

#### Key outputs identified by this workgroup

• Improve descriptive epidemiology of preterm birth

• Improve analytical (risk factor identification) epidemiology

• Strengthen data collection and analysis capacity to inform health care policy

### 5. Stillbirth epidemiology

#### Overarching goal

Stillbirths are an important indicator of women's health, and accurate collection of data will help influence health care providers and policymakers to improve maternal and child health

#### Key outputs identified by this workgroup

• Ensure the collection of comparable data of high-quality, and build capacity

• Develop uniform classification for stillbirths

• Develop targeted and in-depth population based studies

• Inform evidence-based policies and interventions

### 6. Intervention development

#### Overarching goal

Generate knowledge to develop new capacities and strengthen existing capacities to improve birth outcomes

##### Key outputs identified by this workgroup

• Set and disseminate research priorities

• Complete prioritized research and share results

• Strengthen research capacity

### 7. Prioritization of interventions for scaling up

#### Overarching goal

Reduce stillbirths and mortality due to preterm birth through development and application of dynamic processes that engages stakeholders for prioritization of evidence-based and context-specific interventions, delivered with high coverage and equity

#### Key outputs identified by this workgroup

• Advocate use of evidence in prioritization among stakeholders

• Ensure inclusion of preterm birth and stillbirth interventions into existing prioritization processes

• Select best intervention candidates for scale-up in health facilities

• Select best intervention candidates for scale-up in communities and homes

### 8. Community-based strategies and constraints

#### Overarching goal

To achieve the maximum reduction in stillbirths and neonatal deaths due to preterm births by implementing effective community-based approaches at high coverage within the continuum of maternal and newborn care. (We aim to achieve by 2020, a one-third reduction in stillbirths and two-thirds reduction in neonatal mortality due to preterm birth in 68 high mortality countries)

#### Key outputs identified by this workgroup

• Deploy, strengthen and sustain community health workers (CHWs) at scale

• Build capacity of community to identify, promote and monitor actions

• Promote and enhance support structures for CHWs and communities

### 9. Facility-based strategies and constraints

#### Overarching Goal

By 2015 all community members will have timely access to effective, affordable and high quality facility-based maternal, newborn and child health (MNCH) care provided by informed and responsive CHWs as part of an integrated and equitable system to reduce perinatal mortality and morbidity

#### Key outputs identified by this workgroup

• Obtain funding dedicated to a prioritized research agenda

• Provide quality care for all community members attending health facilities

• All facilities provide core packages of MNCH services at first and referral levels—quality facility-based care is accessible to all mothers, newborns and children in a timely manner

### 10. Advocacy and policy

#### Overarching Goal

Key actors allocate sufficient resources and support policies, programs, and actions at the global, regional, national, and community levels to ensure safe full-term pregnancies and healthy newborns

#### Key outputs identified by this workgroup

• Increase funding for research on the scope, causes, consequences, interventions, and scaling-up of interventions for preterm birth and stillbirth

• Increase awareness of the magnitude, impact, and opportunities to reduce and prevent preterm birth and stillbirth, as they relate to the accomplishments of the MDGs

• Build financial and political support for scaling-up a core set of evidence-based, effective interventions for preventing and managing preterm birth and stillbirth, and integrate into national policies and guidelines

### 11. Ethics and social justice

#### Overarching goal

To help inform an ethically responsible and culturally appropriate response to the global burden of preterm birth and stillbirth

#### Key outputs identified by this workgroup

• Identify the range of ethical or social justice considerations that arise along the research pathway, from definitions to discovery, development and delivery

• Engage scientists and key stakeholders regarding the ethical and social justice considerations identified above

• Set a research agenda to address gaps in ethical guidance, policy, and cross-cultural understanding of the ethical issues surrounding the global burden of preterm birth and stillbirth

### 12. Resources and funding

#### Overarching goal

Improve coordination and increase global and national funding for preterm birth and stillbirth within the Reproductive, Maternal, Newborn and Child Health (RMNCH) context

#### Key outputs identified by this workgroup

• Effective international MNCH leadership to influence global health initiatives

• Catalyze, facilitate, leverage and provide targeted support to engage national governments to test and/or scale up effective interventions

• Accelerate progress in the discovery, development, and delivery of low-cost solutions that may be used in all settings

## Post-conference momentum

This collaborative strategy to address preterm birth and stillbirth will also help accelerate improvements in maternal, newborn and child health. Progress on these inseparable outcomes depends on a more coordinated and interdisciplinary approach. GAPPS, participants, and other stakeholders have already begun to work on the following four sets of initiatives:

### Tell the story

• Within the global health community at the highest levels

• Within the broader community, increase awareness and understanding for the magnitude of the problem

### Close the research gaps

• Standardize definitions, classification systems, and data collection

• Accelerate research and alleviate obstacles through increased collaboration

• Accelerate translation of discoveries to interventions

• Improve coordination between intervention development and delivery

• Build in-country research capacity

### Support the discovery, development, and delivery of interventions globally

• Scientists, advocates and funders must work together with countries that have the greatest health burden

### Collaborate to unlock resources

• Includes resources needed for the discovery of what does and does not work, and for the development and delivery of effective interventions

• Inform advocates and funders with a collective, unified voice

• Improve coordination between funders and opportunities

• Increase funding at global and national levels

Ongoing, interdisciplinary dialogue will continue to be fostered by GAPPS. It is important to note that much of the feedback was to move up the deadline for milestones, as much of the work has recently been initiated or is already in progress. Identification of lead agencies and core team members responsible for implementation must also be identified for each output. Examples of new and ongoing activities identified in the GAA will be posted at http://www.gapps.org.

## Authors' contributions

The article was written and reviewed by all authors. The summary section of the Global Action Agenda was based on workgroup recommendations developed by participants during the 2009 International Conference on Prematurity and Stillbirth.

## Competing interests

The authors declare they have no competing interests
